# The Development and Homing of Myeloid-Derived Suppressor Cells: From a Two-Stage Model to a Multistep Narrative

**DOI:** 10.3389/fimmu.2020.557586

**Published:** 2020-10-26

**Authors:** Nathan Karin

**Affiliations:** Department of Immunology, Faculty of Medicine, Technion—Israel Institute of Technology, Haifa, Israel

**Keywords:** CCR5, CCR2 chemokines, cancer, myeloid derived suppressor cells, chemokine

## Abstract

Myeloid-derived suppressor cells (MDSC) represent a heterogeneous population of immature myeloid cells. Under normal conditions, they differentiate into macrophages, dendritic cells, and granulocytes. Under pathological conditions, such as chronic inflammation, or cancer, they tend to maintain their immature state as immature myeloid cells that, within the tumor microenvironment, become suppressor cells and assist tumor escape from immune eradication. MDSC are comprised of two major subsets: monocytic MDSC (M-MDSC) and polymorphonuclear MDSC (PMN-MDSC). Monocytic myeloid cells give rise to monocytic cells, whereas PMN-MDSC share similarities with neutrophils. Based on their biological activities, a two-stage model that includes the mobilization of the periphery as myeloid cells and their activation within the tumor microenvironment converting them into suppressor cells was previously suggested by D. Gabrilovich. From the migratory viewpoint, we are suggesting a more complex setup. It starts with crosstalk between the tumor site and the hematopoietic stem and progenitor cells (HSPCs) at the bone marrow (BM) and secondary lymphatic organs, resulting in rapid myelopoiesis followed by mobilization to the blood. Although myelopoiesis is coordinated by several cytokines and transcription factors, mobilization is selectively directed by chemokine receptors and may differ between M-MDSC and PMN-MDSC. These myeloid cells may then undergo further expansion at these secondary lymphatic organs and then home to the tumor site. Finally, selective homing of T cell subsets has been associated with retention at the target organs directed by adhesion molecules or chemokine receptors. The possible relevance to myeloid cells is still speculative but is discussed.

## Introduction

The tumor microenvironment (TME) is the environment around the tumor that includes sounding blood vessels; immune cells; fibroblasts; soluble mediators, such as cytokines, chemokines, and growth factors; and extracellular matrix (ECM). Among the immune cells that enable tumor escape from immune eradication are myeloid-derived suppressor cells (MDSC). These are a heterogeneous population of cells that consists of myeloid progenitor cells and immature myeloid cells (IMCs). Under nonpathological conditions, these IMCs differentiate into monocytic cells that later become macrophages, dendritic cells (DC), and mature granulocytes. However, under stress and during chronic inflammation, particularly cancer, they tend to response to “emergency signals” (1, 2), and as a result, their maturation into fully differentiated cells is inhibited while retaining their suppressive activity (3–7). Their mechanism of action includes secretion of Arginase 1 (encoded by ARG1) and inducible nitric oxide synthase (iNOS, also known as NOS2) as well as an increase in their production of nitric oxide (NO) and reactive oxygen species (ROS) [for a recent review, see (8)]. MDSC also express immune checkpoint inhibitors, among them PD-L1 and also PD-1 (9). Along with this, very recently it has been reported that targeted deletion of PD-1 from MDSC induces highly effective antitumor immunity (10). Altogether, these render MDSC immune suppressive, in particular of effector T cells, which enables tumor escape from immune eradication (3–7). It is, thus, believed that these cells play a major role in enabling tumors to escape their elimination or blockade, which could be beneficial for cancer immunotherapy (11).

**Figure 1:**
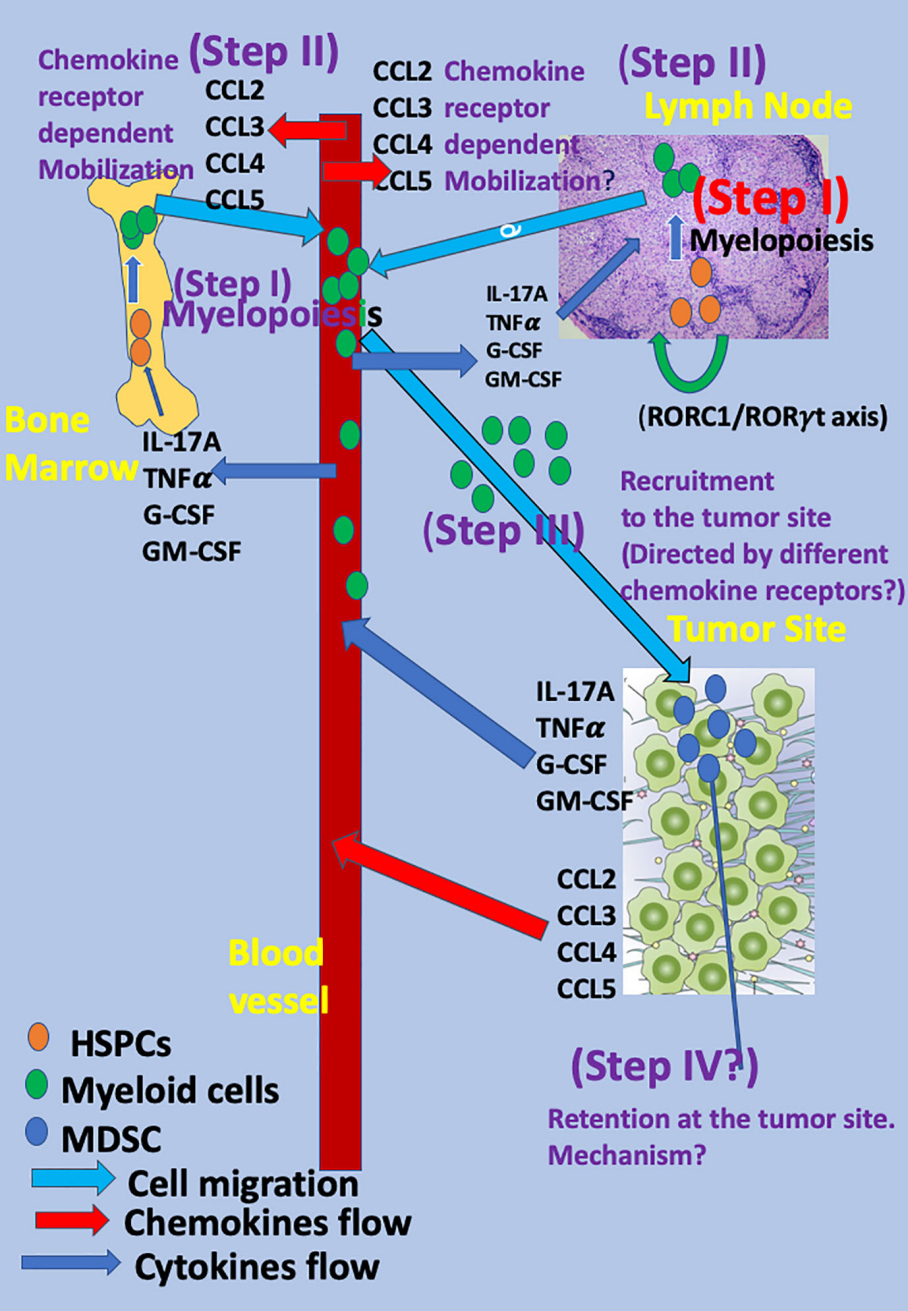
*The mobilization and migration of myeloid cells to the tumor site as a multistep event* The mobilization and migration of myeloid cells to the tumor site is a multistep event in which cytokines, chemokines, and transcription factors released from the tumor site reach the blood and, thereafter, the BM and LNs and direct the different steps in myeloid cell differentiation and migration. The first step (Step I) is rapid myelopoiesis of myeloid cells at the BM and secondary lymphatic organs (LNs and spleen) and is directed by several cytokines, among them interleukin-17A (IL-17A), G-CSF, GM-CSF, TNFα, and others. Recently, the key role of the retinoic acid–related orphan receptor (RORC1/ROR/γ) in directing myelopoiesis in LNs has been observed (2). The subsequent step (Step II) includes the mobilization of myeloid cells to the blood and is directed by specific chemokine receptors: CCR2 for monocytic myeloid cells (15) and CCR5 for the polymorphonuclear myeloid cells (16) *via* CCR2 key ligand CCL2 and the CCR5 key ligands: CCL3, CCL4, and CCL5 (Step II). Homing to the tumor site is likely to be directed by many chemokines and chemokine receptors and is likely to have low specificity (Step III). Step IV includes the retention of these cells at the tumor site and, thus far, has been mostly studied for T cells (17–20). For myeloid cells, it is still speculative.

Myeloid cells, as other bone marrow (BM)-derived cells, are generated from hematopoietic stem and progenitor cells (HSPCs) in a process termed myelopoiesis and then are mobilized from the BM to the blood. HSPCs also migrate from the BM to secondary lymph nodes and spleen (12). At these organs, the presence of myeloid cells has also been recorded [reviewed in (13)]. Recently, it has been reported that under “emergency” conditions occurring during stress, inflammation, and cancer diseases, the retinoic acid–related orphan receptor (RORC1/ROR/γ) orchestrates emergency myelopoiesis by suppressing negative (Socs3 and Bcl3) and promoting positive (C/EBPb) regulators of granulopoiesis as well as the key transcriptional mediators of myeloid progenitor commitment and differentiation to the monocytic/macrophage lineage (IRF8 and PU.1) (2). This may suggest that, under emergency conditions, myelopoiesis and rapid extension of myeloid cells may also take place at secondary lymphatic organs and spleen and, by so doing, allow massive accumulation of these cells at tumor sites (2) [(see also reviews in ((1) and (14)) (**Figure 1**)].

Chemokines are a subgroup of chemotactic cytokines that are well associated with chemo-attraction of various leukocytes, either from the BM to the blood (mobilization); from the blood to sites of inflammation, autoimmune sites, tumor sites, etc.; and from tissues and blood to the lymph nodes (21–23). The current review focuses on elaborating a sequential multistep model for characterizing their myelopoiesis, mobilization, recruitment, retention, and biological function. In this model, the migratory properties of myeloid cells from BM (and perhaps also from secondary lymphatic organs) to the blood (mobilization), is likely to be directed by specific chemokine receptors (**Figure 1**). The model that we are suggesting does not contradict the two-stage model of Gabrilovich (11), but adds several steps that are associated with the migratory properties of these cells. For example, the first step in Dr. Gabrilovich’s model corresponds to activation of myelopoiesis, mobilization to the blood, and migration of myeloid cells to the tumor sites as suggested in our multistep model as different steps.

## MDSC Subtypes

MDSC are comprised of two major subsets: monocytic MDSC (M-MDSC), and polymorphonuclear MDSC (PMN-MDSC). In human, M-MDSC are defined as CD11b+ CD14+ CD15−HLA-DRlow/− cells. Due to the low or absent HLA-DR expression, M-MDSC can be distinguished from monocytes. Human PMN-MDSC are characterized as CD11b+ CD14−CD15+ HLA-DR− or CD11b+CD14−CD66b+ (24).

In mice, M-MDSC are defined as CD11b+Ly6G−Ly6C^high^ and share phenotypical and morphological characteristics with monocytes. PMN-MDSC are described as CD11b+Ly6G^high^Ly6C^low^ cells and resemble neutrophils (24).

M-MDSC and tumor-associated macrophages (TAMs) share many features (25). Thus, it is believed that, at the tumor site, M-MDSC may become TAMs. The question of whether PMN-MDSC may also become mature granulocytes is still an open question. There are two lines of evidence that support this hypothesis: 1. Tumor-associated neutrophils and G-MDSC represent similar functional states of cells originating from the same cell type and induced within a tumor host. 2. Neutrophils isolated from a normal tumor-free host substantially differ from tumor-associated neutrophils or G-MDSC obtained from a tumor-bearing host [reviewed in (26)].

Both types of MDSC express many chemokine receptors, among them CCR5 and CCR2 (27). Within the TME, a vast majority of MDSC are PM-MDSC (about 80%) even though they have a shorter lifetime (11). Both also operate *via* similar mechanisms of immunosuppression with a few differences: Arginase-1 and prostaglandin E2 (PGE2) are preferentially produced by PMN-MDSC, whereas NO is by M-MDSC [for a recent review, see (3)]. It is also believed that M-MDSC are more prominent than PMN-MDSC as they are thought to rapidly differentiate to TAMs at the tumor site (28–35), whereas PMN-MDSC play a major role in inducing peripheral T cell tolerance (3, 11).

## The Two-Stage Model of Myeloid Cells Mobilization and Function

Myelopoiesis during acute infection, stress, or trauma results in rapid terminal differentiation of myeloid cells. By contrast, in cancer and chronic inflammation, myelopoiesis is associated with defective myeloid cell differentiation, which results in the accumulation and persistence of immature myeloid cells at cancer sites or chronic inflammatory sites. These cells then function as suppressor cells and are, therefore, referred to as MDSC (4, 6–8). Based on the above, D. Gabrilovich et al. suggest a two-stage model that is based on the biological function of myeloid cells during cancer and chronic inflammation (11). It includes the myelopoiesis of these cells in BM, their mobilization to the blood and secondary lymphatic organs as myeloid cells (stage I), and later their transition and maintenance as MDSC (stage II), which mostly takes place at the tumor site (11) or, respectively, sites of chronic inflammation (36).

In both type of diseases, the rapid myelopoiesis of myeloid cells at the BM is likely to be directed by several cytokines and transcription factors, among them interleukin-17A (IL-17A) ROR1C that induces IL-17A, G-CSF, GM-CSF, TNF*α* and others (2, 4, 6, 14, 37, 38), whereas maintenance of the suppressive function is driven by several components that affect the activities of MDSC at the tumor site, including interaction with other cells, particularly T cells cytokines, chemokines, and transcription factors, and the effect of microRNA released from exosomes (39–41).

The second stage includes two distinct yet partially overlapping types of signals. The first is associated with the expansion of the immature myeloid cells and inhibition of their terminal differentiation, and the second is their pathologic activation as suppressor cells (42). The first group of signals is mostly driven by tumor-derived growth factors as well as STAT3, IRF8, C/EBPb, Notch, adenosine receptors A2b signaling, and NLRP3 (43) and of microRNA released from exosomes (39–41). The second type of signal is mediated by factors produced mostly by the tumor stroma (proinflammatory cytokines, HMGB1) and includes the NF-kB pathway, STAT1, STAT6, prostaglandin E2 (PGE2), and cyclooxygenase 2 (COX2) as reviewed in (42).

It should be noted that the mechanisms controlling the suppressive activities may vary between PMN-MDSC and M-MDSC. The first are short-lived (44), and their activity may require close cell-to-cell contact with T cells (45), whereas M-MDSC are long-lived and are likely to give rise to TAMs that, under the TME milieu, suppress antitumor immune reactivity by different mechanisms (46).

Despite the clear definition between myeloid cells in the periphery and MDSC at the tumor site (11), it has been reported in cancer MDSC in spleen, and secondary lymphatic organs function as suppressor cells and execute far-reaching immune suppression by reducing expression of the L-selectin lymph node (LN) homing receptor on naive T and B cells, and impair T cell activation also by inhibiting the homing of naïve CD4+ and CD8+ T cells to LNs (47).

## The Recruitment of MDSC at Tumor Sites as a Multistep Event Directed in Part by Chemokine–Chemokine Receptor Pathways

The generation of myeloid cells and their recruitment to the tumor site could be viewed as a multistep event, in which the cross-talk between the tumor site and myeloid cells play a major role. We are suggesting a four-step event that characterizes the homing of these cells (step I–IV) and an additional two steps that aim to focus on two complementary signaling events within the TME that enable the transformation of myeloid cells into suppressor cells and maintains their immature state as such (**Figure 1**) as follows:

### Step I

The first step is myelopoiesis. It could occur in the BM and also possibly in the LNs and spleen as HSPCs also migrate from BM to LNs, spleen, and peripheral tissues (12) and undergo myelopoiesis there (2). Several key cytokines take a major role in this step, including IL-17A, granulocyte-colony stimulating factor (G-CSF), granulocyte-macrophage colony-stimulating factor (GM-CSF), and TNFα. All these cytokines are largely produced at tumor sites, and their blood levels increase during cancer diseases (48–56). Concurrent myelopoiesis at the BM and secondary lymphatic organs may allow intensive accumulation of myeloid cells at the tumor site (1, 2, 14).

### Step II

The subsequent step (step II) is the mobilization of myeloid cells that rapidly proliferated along myelopoiesis from the BM and possibly secondary lymphatic organs to the blood. It is not yet clear whether the mechanism by which these cells are mobilized from the BM to the blood differs from the one by which they are mobilized from the lymph nodes to the blood. Accumulating evidence votes for a pivotal role of chemokine–chemokine receptor interactions at this step (15, 16, 57). Several key chemokines are largely produced at tumor sites, and their blood level increases during cancer diseases, among them the CCR2 ligand CCL2 (58), and CCR5 ligands, in particular CCL5 (59, 60). These soluble mediators are likely to participate in the inter-talk between the tumor and leukocytes, either within the tumor site or at the periphery. The CCR2–CCL2 axis is highly relevant for monocytic and monocytic myeloid cells (15, 57), particularly in inflammation (15) and cancer (28–31, 33, 34). In 2003, Geissmann, Jung, and Littman reported different migratory properties for CX3CR1^low^CCR2+Gr1+ and CX3CR1^high^CCR2-Gr1+ cells, showing that those that are CCR2+ preferentially home to inflammatory sites, whereas the others go to normal tissues (57). This links CCR2 to selective homing of monocytic myeloid cells to inflammatory sites (57). Three years later, Sebrina et al. demonstrated the pivotal role of CCR2 in directing the mobilization of Ly6C^high^ monocytes from BM to the blood (15). This study shows that CCR2KO mice display fewer circulating Ly6C^high^ monocytes and, after infection with listeria monocytogenes, accumulate activated monocytes in BM (15). This study also shows that the later chemotaxis of these cells to the inflammatory site is not necessarily CCR2-dependent and also occurs if using monocytic cells from CCR2 KO mice (15). Later studies further explore the pivotal role of CCR2 in directing the recruitment of CCR2+ monocytic cells to the tumor site to support its development and suppress antitumor immunity (28–31, 33, 34). More recently, Chang et al. observed in murine glioma that CCL2 produced by microglia recruited CCR2+Ly6C+ monocytic MDSCs (M-MDSCs) to the tumor site, which is absent in CCR2KO mice (61). Among the different ligands that bind CCR2, CCL2 has been thought to be the dominant chemokine. An additional chemokine that is likely to hold similar properties is CCL12 (62).

Less is known about the mobilization of polymorphonuclear myeloid cells from the BM (and perhaps lymphatic organs) to the blood. Our group found interest in exploring the role of CCR5 and its ligands in cancer. Individuals with a functional mutation in CCR5 (deletion of the N-terminal 32 nucleotides) display a high state of HIV resistance (63). Later, it was found that they also show low prevalence of cancer diseases, particularly cancer of the prostate (64). This motivated us to explore the underlying mechanism by which the absence of CCR5 confers cancer resistance. In so doing, we have used CCR5KO mice and an autologous model of prostate cancer in immunocompetent mice (16). In this study, we observed that 1. CCR5 ligands directly support tumor growth *via* CCR5, and thus, blockade of CCR5 ligands in a chimeric system in which CCR5KO mice bearing CCR5+ tumor cells, targeting CCR5 ligands restrains tumor growth (16). 2. CCR5 drives the accumulation of MDSC at the tumor site; thus, in CCR5KO mice, the relative number of GR1+ CD11b+ myeloid cells at the tumor site is very low, and tumor development is arrested. Reconstitution of these mice with GR1+ CD11b+ myeloid cells from WT mice (CCR5+) reconstituted tumor growth (16). Further investigation shows that, along with tumor development the level of CCR5 ligands that are largely expressed with the TME, increases in the blood. This leads to a rapid increase in the expression of CCR5 on myeloid cells at the BM to a rapid mobilization of CD11b+Ly6G^high^Ly6C^low^ myeloid cells that become PMN-MDSC at the tumor site (16). It has yet to be studied if limited accumulation of PMN-MDSC at the tumor site in CCR5KO mice exclusively results from reduction in their mobilization from the BM to the blood, and/or from secondary lymphatic organs to the blood, or also due to possible roles of CCR5 in directing the accumulation of these cells at the tumor site. In this study, we also observed that the CCR5–CCR5 ligands interaction also potentiates the suppressive activities of PMN-MDSC by increasing the expression of Arginase 1 and possibly other mediators that suppress effector T cell function (16). A recent manuscript used the technology of deleting the genomic locus incorporating the iCCRs of different chemokine receptors that have been associated with myelomonocytic cell population migration, including CCR1, CCR2, CCR3, and CCR5 to show that tissue-resident myelomonocytic cell populations are established even in their absence, whereas during inflammation, CCR2 holds a key role in their targeted recruitment (65). Dyer et al. have not explored their setup in a cancer disease model.

### Step III

The third step (step III) includes the accumulation of MDSC at the tumor site and their retention there. In our opinion, this step is more complex and less understood than most leukocyte subtypes. The major obstacle is that myeloid cells express many different chemokine receptors and may, thus, respond to many different chemokines that are largely expressed at tumor sites. Then, what causes chemokine receptor specificity? Indeed, many studies show a significant role of different chemokines in myeloid cell recruitment to tumor sites (**Table 1**). Among them, 1. the CCL15-CCR1 signaling pathway (68, 69), 2. the CX3CL1 - CCL26 pathway for recruiting M-MDSC (70), 3. the CXCL5/CXCL2/CXCL1 chemokines were suggested to recruit PMN-MDSCs to tumor tissue *via* CXCR2 in murine spontaneous melanoma model (71), 4. CXCL13-CXCR5 signaling mediates the migration of MDSCs to tumor tissue (72). Moreover, in different cancer diseases, poor or good prognosis was associated with high or low levels of these chemokines (**Table 2**) [for a recent review, see (73)]. How could these observations take place with the predominate role of the CCR5-axis for directing PMN-MDSC recruitment and the CCR2-axis for M-MDSC selective recruitment at tumor sites? We are suggesting, within the three-step model described above, the highly selective step that determines receptor specificity is the mobilization from the BM and perhaps from the secondary LNs to the blood and that this step serves as a bottleneck for selectivity in myeloid cell migration.

**Table 1:** The role of chemokines, cytokines, and other mediators in directing the different steps in myeloid cell migration and function.

Step	Mediators	References
Step I: Myelopoiesis	IL-17A, G-CSF, GM-CSF, TNFα, RORC1,	(1, 2, 43, 66, 67)
Step II Mobilization to the blood (and possibly also homing to the tumor site):	CCR2 ligands (mostly CCL2) for monocytic cells, and CCR5 ligands, preferentially CCL5 for PMN-MDSC	(15, 16, 57)
Step III: Homing to the tumor site	CCL15-CCR1 signaling pathway, CX3CL1 - CCL26 pathway, the CXCR2-CXCL5/CXCL2/CXCL1 pathway, the CXCL13-CXCR5 pathway	(68–72). Also recently reviewed in (73)
Step IV: Retention at the tumor site	Firm data only for T cells. Yet to be identified for myeloid cells.	For T cells: (17–20, 74–76),
expansion of the immature myeloid cells and inhibition of their terminal differentiation at the tumor site	STAT3, IRF8, C/EBPb, Notch, adenosine receptors A2b signaling, and NLRP3 and of microRNA released from exosomes	(39–41, 43)
Transformation of the immature myeloid cells into suppressor cells	proinflammatory cytokines HMGB1, STAT1 STAT6, prostaglandin E2 (PGE2) cyclooxygenase 2 (COX2)	reviewed in (42)

**Table 2:** Key chemokines associated with myeloid cell homing and cancer prognosis.

****Chemokine	****Key Target receptor	Step****	****Association with prognosis in the following cancer diseases	****Reference
CCL2	CCR2	II III?	Pancreatic cancer, Bladder cancer, Breast cancer, Lung Adenocarcinoma, Renal cell carcinoma, Ovarian cancer, Cervical carcinoma	(77–83)
CCL5	CCR5	II III?	Breast cancer, Glioblastoma, Colorectal cancer, Osteosarcoma, Gastric cancer, Hepatocellular carcinoma	(59, 60, 84–91)
CCL15	CCR1	III	Head and Neck Squamous Cell Carcinoma (HNSCC), Colorectal cancer, Gastric cancer, Hepatocellular carcinoma, Lung cancer	(69, 92–97)
CCL26	CX3CL1	III	Colorectal cancer	(98)
CXCL5/CXCL2/CXCL1	CXCR2	III	Pancreatic ductal adenocarcinoma, Glioblastoma, Non-small cell lung cancer, Gastric Cancer, Prostate cancer, Colorectal cancer, Bladder cancer	(99–107)
CXCL13	CXCR5	III	Clear Cell Renal Cell Carcinoma (ccRCC), Gastric cancer, HBV-related hepatocellular carcinoma, Breast cancer, Lymphoma	(108–114),

### Step IV

Retention at the tumor site: This step is still speculative and has been mostly studied for T cells thus far. It suggests that, tentatively, myeloid cells could be recruited to tumor sites by many different chemokine receptors, but their retention there is more specific and may involve a limited number of chemokine receptors and/or adhesion molecules (74). This option has been explored thus far only for T cell migration. Key examples are the retention of CD103+ memory T cells to tissues where they become tissue resident memory T cells due to the interaction of CD103 (an αE integrin) that binds a β7 counter integrin (17–20), the L-selectin serving as a homing receptor for naïve T cells (75), and the role of the α4β1 integrin in the retention of CD4+ T cells in the inflamed brain (76). The relevance of this concept to other leukocyte subtypes is yet to be studied. 

## Post-Translational Modification (PTM) of Chemokines and Selective Migration of PMN-MDSC

An important mechanism of fine-tuning chemokine activity is PTM of chemokines and their receptors. One of the mechanisms that may show high relevance to CCR5-dependent selective migration of PMN-MDSC is PTM by CD26 [for a recent relevant review, see (115)]. CD26 is a cell-bound enzyme ubiquitously expressed on blood cells, especially on activated T cells, fibroblasts, and epithelial cells. Two of the three CCR5 ligands, CCL4, and CCL5 are truncated by CD26, which may selectively reduce CCL4/CCL5 activity on T cells but to a lesser degree extend PMN-MDSC.

## Clinical Implications in Cancer Diseases: Could Redundancy in Chemokines Be Overcome *via* Monotherapy?

Thus far, many clinical trials in humans in which single chemokines or their receptors were targeted for therapy of inflammatory autoimmunity or cancer failed. Two major potential reasons could be taken into account: redundancy, that is, different chemokines with similar properties bind the same chemokine receptor, and overcompensation by production of increased levels of targeted chemokine. Two possible approaches to partially overcome this obstacle is by designing a compound that would target all ligands of a single receptor or prefer a receptor blockade over targeting a single chemokine. We have taken the first approach and generated a chimeric CCR5 soluble receptor study (116) that could effectively inhibit cancer of the prostate in C57Bl/6 mice (16). Then, together with Viktor Umansky and his team, this study was further extended to a transgenic model of melanoma showing that indeed the CCR5-CCR5 ligand axis directs the accumulation of PMN-MDSC at the tumor site and that CCR5-Ig also effectively inhibited the development and progression of this disease (117). Others used blocking mAbs to CCR5 or even one of its three ligands, CCL5, to inhibit metastasis and improve the survival of tumor-bearing mice (118, 119) and also enhance anti-PD1 efficacy in gastric cancer (120). As for humans, Halama et al. recently showed success in blocking colorectal cancer using a CCR5 small molecule inhibitor that was previously developed for therapy of HIV (121). If successful, we think that extension of this therapeutic approach as a monotherapy or part of a combined therapy could be further considered.

## A Future View of the Classical Two-Stage Model in Light of Modern Technologies

The traditional classification of myeloid cells in the periphery and MDSC at the tumor site have recently been revised using several modern technologies, aiming at categorizing single cells based on their gene signature (single-cell RNAseq) and expression of cell surface receptors and some intracellular proteins (mass cytometry, CyTOF). The basic hypothesis is that, beyond the variety between human and mouse in the classification of these cells (24), in each species, the gene signature and cell surface protein expression may vary depending on the organ from which cells are isolated (BM, spleen, blood, LNs, tumor site) and may also differ between tumor types (122–126). These studies are still in early development but may pave a new direction in scientific research and its translational implications.

## Can the Multistep Model Explain the Paradox of Redundancy in Chemokine–Chemokine Receptor Interactions and Selective Migration?

MDSC express many chemokine receptors and may, therefore, potentially migrate in response to each of them (**Table 1**). The multistep model suggests that, among the four different steps (myelopoiesis, mobilization to the blood, recruitment, and retention) the step of mobilization to the blood is likely to be the more highly selective stage. In this event, CCL2 signals *via* CCR2 to allow the effective mobilization of monocytic cells, including monocytic myeloid cells (15, 57), whereas CCR5 *via* its ligands, mostly CCL5, is likely to direct the mobilization of PMN myeloid cells (16). The last has mostly been studied in our laboratory and has to be further confirmed by others. It is conceivable that the CCR2 and CCR5 axes are also involved, together with other axes in step III of homing to the tumor site (**Table 1**). This may explain why CCR2 and perhaps CCR5 are indeed key drivers in the migratory cascade of myeloid cells.

Among these four steps, not much is known for the last one (retention) for myeloid cells. For T cells, its selectivity and specificity are mostly directed by selective adhesion receptors (18–20, 75, 76). We do not exclude the possibility that a key adhesion molecule or a key chemokine receptor may direct this stage, making it a highly selective step as well.

## Conclusions

Based on their biological function, the development of MDSC includes two major stages: the first starts with myelopoiesis in the BM and lymphatic organs and the second upon their entry to the tumor site where they acquire suppressive capabilities and retain their amateur state of development. Nevertheless, based on their migratory properties, their generation and migration to the tumor site could be described as a more detailed multistep event in which their mobilization to the blood seems to be chemokine-receptor dependent and also determines the selectivity of their migration. We have uncovered a key role of the CCR5 axis in directing the mobilization of PMN-MDSC and suggest CCR5 blocking as a potential way for monotherapy or part of combined therapy for cancer diseases.

## Author Contributions

The author confirms being the sole contributor of this work and has approved it for publication.

## Funding

This study was supported by the Ministry of Science & Technology of the state of Israel (MOST) and the German Cancer Research Center (DKFZ) (MOST- DKFZ grant # 3-16001 and grant # GR-2471) Israel Cancer Research fund (ICRF) (PG-19-105 and PG-171961), Israel Cancer Association (ICA 2020-1209), Collek Research fund, and Israel Science Foundation (ISF, 283/19).

## Conflict of Interest

The author declares that the research was conducted in the absence of any commercial or financial relationships that could be construed as a potential conflict of interest.
